# No Association of Polymorphisms in the Genes Encoding Interleukin-6 and Interleukin-6 Receptor Subunit Alpha with the Risk of Keloids in Polish Patients

**DOI:** 10.3390/ijms25105284

**Published:** 2024-05-13

**Authors:** Andrzej Dmytrzak, Klaudyna Lewandowska, Agnieszka Boroń, Beata Łoniewska, Natalie Grzesch, Andrzej Brodkiewicz, Jeremy S. C. Clark, Andrzej Ciechanowicz, Dorota Kostrzewa-Nowak

**Affiliations:** 1 Aesthetic Med, 71-403 Szczecin, Poland; aestheticmed@aestheticmed.com.pl; 2 Department of Clinical and Molecular Biochemistry, Pomeranian Medical University, 70-111 Szczecin, Poland; klaudyna.lewandowska@pum.edu.pl (K.L.); agnieszka.boron@pum.edu.pl (A.B.); natalie.grzesch.ng@gmail.com (N.G.); jeremy.clark@pum.edu.pl (J.S.C.C.); andrzej.ciechanowicz@pum.edu.pl (A.C.); 3 Department of Neonatal Diseases, Pomeranian Medical University, 70-111 Szczecin, Poland; beata.loniewska@pum.edu.pl; 4 Department of Pediatrics, Child Nephrology, Dialysotherapy and Management of Acute Poisoning, Pomeranian Medical University, 70-780 Szczecin, Poland; andrzej.brodkiewicz@pum.edu.pl

**Keywords:** keloid, genetic associations, *IL6*, IL-6, *IL6R*, IL-6RA

## Abstract

A keloid is a benign fibroproliferative hypertrophy of scar tissue that extends outside the original wound and invades adjacent healthy skin. Keloid formation is thought to be a complex process including overactivity of the interleukin-6 signaling pathway and genetic susceptibility. The aim of the study was to investigate possible associations between rs1800797, rs1800796, and rs1800795 polymorphisms in the promoter of the *IL6* gene encoding interleukin-6 and the rs2228145 polymorphism in the *IL6R* gene encoding the interleukin-6 receptor subunit alpha with the predisposition to keloids in Polish patients. The genetic polymorphisms were identified either using Polymerase Chain Reaction-Restriction Fragment Length Polymorphism (PCR-RFLP) or sequencing of samples of genomic DNA extracted from blood leukocytes of 86 adult patients with keloids and 100 newborns comprising a control group. No significant differences in the distributions of *IL6* or *IL6R* alleles or genotypes were found between keloid patients and newborn controls. There were also no significant differences between both groups in the distribution of *IL6* haplotypes. The *IL6* rs1800797, rs1800796 and rs1800795 and *IL6R* rs2228145 polymorphisms were not found to predispose individuals in the study group to keloids. *IL6* promoter haplotypes were not found to be associated with a higher risk of keloids in the studied group.

## 1. Introduction

A keloid is benign, locally aggressive, fibroproliferative hyperplasia of scar tissue, classified as a benign skin tumor that extends outside the original wound and invades healthy skin. Unlike hypertrophic scars, which usually do not exceed the borders of the scar and tend to regress spontaneously, a keloid may grow beyond its borders, and spontaneous regression is rarely observed [1]. The first available description of a keloid (or perhaps a hypertrophic scar) was included in the Edwin Smith Papyrus from approximately 3600–3700 years ago [2]. The word “keloid” in the English language comes from the term cheloide, a combination of two Greek words chele (crab claws) and oid (similar) first used in 1790 by the French physician Noël Retz and described in 1816 by Jean Louis Alibert [3,4,5].

The etiology of keloids and the pathomechanism of their formation are still not fully understood [6]. It is thought that the formation of a keloid scar is a complex process that depends on many factors, and the risk factors for its formation include wound infection, skin tension, race, immune system disorders, and genetic susceptibility. Keloids can occur in both women and men, and the groups at increased risk of keloid formation include pregnant women, teenagers, and young adults. Keloid occurrence also affects all major ethnic groups, but it is most often found in individuals of relatively recent African descent (e.g., African-Americans) and in people of Asian descent [1,6,7]. In subjects with dark skin color, keloids are detected approximately 4–16 times more often than in people of European descent [1,6,7]. It should also be emphasized that no cases of keloid scarring have been confirmed in albinos [8]. Much evidence indicates that genetic factors play an important role in the pathogenesis of keloid scarring. In addition to the above-mentioned ethnic-dependent differences in keloid prevalence [1,6,7], the most important evidence pointing to the genetic susceptibility to keloid formation comes from familial occurrence, the identification of chromosomal loci linked to keloids in some families, as well as the identification of genetic polymorphisms predisposing to the development of keloids (from genome-wide association studies or from association studies focused on individual candidate genes) [9,10,11,12,13,14,15,16,17,18,19,20,21,22]. An additional argument confirming the role of hereditary factors in the pathogenesis of this disease is the occurrence of keloids in some monogenic diseases (e.g., vascular-type Ehlers–Danlos syndrome caused by *COL3A1* mutations or Rubinstein–Taybi syndrome-1 caused by *CREBBP* mutations) [1,23,24].

Excessive activation of interleukin-6-dependent signal transduction seems to play an important role in the pathogenesis of keloid scarring [25]. In 2005, Tosa et al. discovered that the expression of IL-6 (both as mRNA and protein) in fibroblasts derived from keloid scars was higher compared to its expression in normal skin fibroblasts [26]. Interleukin-6 (UNIPROT short name: IL-6, P05231) is one of the main cytokines involved in the immune response to skin injury [14]. Interleukin-6 signal transduction into the cell requires the participation of two molecules: an α subunit (ligand-binding subunit) of 80 kDa that is the proper receptor for this cytokine (UNIPROT: IL-6RA; P08887) and the β subunit (signal transduction subunit), a 130 kDa glycoprotein (UNIPROT: IL-6RB; alternative name: gp130). The interleukin-6 ligand first binds to IL-6RA, and only after binding does IL-6/IL-6RA combine with IL-6RB, forming a stable complex that, after homodimerization, becomes capable of transmitting a signal [27].

The *IL6* gene that encodes interleukin-6 is located on chromosome 7p21 and, three single nucleotide polymorphisms (SNPs) have been identified in its promoter (region −600 to −1 from the translation initiation site): rs1800797 (−597G > A), rs1800796 (−572G > C), and rs1800795 (−174G > C) [28].

The rs2228145 polymorphism in the *IL6R* gene, encoding the interleukin-6 receptor subunit alpha (IL-6RA), consists of a transversion of adenine to cytosine at position 1073 (c.1073A > C). This results in the substitution of aspartic acid with alanine at position 358 of the IL-6 alpha receptor polypeptide chain (p.Asp358Ala) at a possible cleavage site of this protein by the metalloproteases ADAM-TS 10 and ADAM 17. The reaction catalyzed by these proteases produces a biologically active extracellular domain of the receptor (sIL-6RA, the soluble form of IL-6RA) that is secreted into circulation. The soluble form of IL6RA can bind to free interleukin-6 present in the blood and other fluids, forming a biologically active complex [29,30].

In 2016, Tosa et al., in a study analyzing *IL6* rs1800797, rs1800796, and rs1800795 and *IL6R* rs2228145 in Japanese subjects, found a significantly higher frequency of the rs1800796:-572G allele in patients with keloid scars compared to healthy subjects. For the two other *IL6* promoter polymorphisms, all individuals studied had the reference homozygous genotype, i.e., GG. However, for *IL6R* rs2228145, the authors did not find significant differences in the distribution of genotypes and alleles between patients with keloid scars and individuals in the control group [13]. The following year, Zhu et al., analyzing only the *IL6* polymorphisms rs1800796 and rs1800795, replicated the finding in Chinese individuals, demonstrating that the rs1800796: −572G allele was associated with a predisposition to keloid formation. In addition, in patients with keloids, the concentration of interleukin-6 in GG homozygotes was significantly higher compared to individuals with the CC homozygous genotype [14]. These observations regarding the association of the *IL6* rs1800796:G allele with a predisposition to keloid formation and serum interleukin concentrations were also replicated in 2019 in a study conducted on a small group of Egyptians (60 patients with keloid scars and 30 healthy Egyptians as the control group) [17].

This raises the question of whether the rs1800797, rs1800796, and rs1800795 polymorphisms in the *IL6* gene promoter or the functional rs2228145 polymorphism of the *IL6R* gene predispose patients of European descent to keloid formation. To address this issue, we decided to investigate the potential associations of these four SNPs with the risk of keloid occurrence in Polish patients.

## 2. Results

The *IL6* rs1800797, rs1800796, and rs1800795 and *IL6R* rs2228145 genotype distributions in the combined group of keloid patients and control newborns conformed to the expected Hardy-Weinberg equilibria (*p* = 0.364, *p* = 0.302, *p* = 0.368, and *p* = 0.674, respectively). No deviations from Hardy–Weinberg equilibria were also observed for the *IL6* or *IL6R* genotypes in the analysis carried out separately for the keloid group (*p* = 0.892, *p* = 0.205, *p* = 0.932, or *p* = 0.858 for *IL6* rs1800797, rs1800796, and rs1800795 and *IL6R* rs2228145, respectively) or for controls (*p* = 0.619, *p* = 0.523, *p* = 0.408, or *p* = 0.889 for *IL6* rs1800797, rs1800796, and rs1800795 and *IL6R* rs2228145, respectively). There were no significant differences in the frequency distributions of *IL6* rs1800797, rs1800796, and rs1800795 and *IL6R* rs2228145 alleles between keloid patients and newborns constituting the control group. The analyses carried out using univariate logistic regression revealed no significant associations between *IL6* rs1800797, rs1800796, and rs1800795 and *IL6R* rs2228145 polymorphisms and the susceptibility to keloids in additive, dominant, or recessive modes of inheritance of their minor (risk) alleles (Table 1).

There was significant (*p* < 0.01) linkage disequilibrium (LD) between each pair of *IL6* promoter polymorphisms (rs1800797-1800795, rs1800797-rs1800796). However, very tight linkage disequilibrium was found only for *IL6* rs1800796-rs1800795 polymorphisms (D` = 0.978, r^2^ = 0.916, *p* = 2.22 × 10^−^^16^). The results of the linkage disequilibrium analysis were used to reconstruct *IL6* haplotypes. The LD-based reconstruction of *IL6* rs1800797-rs1800796-rs1800795 haplotypes revealed a lack of the A-C-G variant (H8 haplotype; haplotypes defined in Table 2). The H1 haplotype (G-G-G) with the highest prevalence, both in the keloid group and in controls, was treated as the reference in further statistical analyses. In addition, three haplotypes (H5, H6, and H7) with a frequency less than 2.0% in the combined group were analyzed together as the rare haplotypes. There were no significant differences in frequency distribution of *IL6* haplotypes between the keloid patients and the control group in the sex-adjusted logistic regression in additive, dominant, or recessive modes of inheritance for the haplotypes in comparison to the reference haplotype (Table 2).

## 3. Discussion

This report focuses on the relationship between the polymorphisms rs1800797, rs1800796, and rs1800795 in the promoter of the *IL6* gene encoding for interleukin-6 and polymorphism rs2228145 of the *IL6R* gene encoding for the alpha subunit of the IL-6 receptor with the predisposition to keloid scar formation in Polish individuals.

Interleukin-6 (IL-6) was discovered in 1986 as a factor that stimulated B lymphocytes and initiated their production of immunoglobulin G antibodies [31]. In the human *IL6* gene, in the first 1200 base pairs upstream from the transcription initiation site, several sequences recognized by transcription factors (cis elements) have been identified [32]. Polymorphisms in the promoter of the *IL6* gene that determine individual differences in transcription regulation and gene expression, and thereby the intensity of cytokine production, shape the predisposition to certain diseases [32]. According to the literature, the four main single nucleotide polymorphisms of the *IL6* gene promoter that collectively influence the transcription of this gene are −2954G > C, −1363G > T, −597G > A (rs1800797), and −572G > C (rs1800796) [32,33,34,35]. The *IL6* rs1800795 (−174G > C) polymorphism is located in what is known as the negative regulatory domain (region −225 to −164) near the cAMP response element (CRE). It is also noteworthy that this polymorphism is located in a sequence partially homologous to the MAD homolog 4 (=Smad4) binding element, and, compared to the reference allele (−174G), the −174C allele binds more effectively to MAD homolog 4, thereby more strongly inhibiting the transcription of the interleukin-6 gene [32].

In contrast to results by Tosa et al. [13], we did not demonstrate a relationship between any of these genetic variants and the risk of keloid occurrence. However, it should be noted that Tosa et al. identified only reference GG genotypes for two of the three analyzed *IL6* polymorphisms, i.e., rs1800797 and rs1800795, both in the study group and in the control group, which is fully consistent with the results of the 1000 Genomes Project (1KG Project) (http://www.ensembl.Org/Homo_sapiens/Variation/Population?db=core;r=7:22726102-22727102;v=rs1800797;vdb=variation;vf=729516893 and http://www.ensembl.org/Homo_sapiens/Variation/Population?db=core;r=7:2272652622727526;v=rs1800795;vdb=variation;vf=729516845, both accessed on 13 March 2024) for Japanese and for three Chinese groups (Han Chinese from Beijing, Han Chinese from Southern China, and the Dai minority from Xishuangbanna Prefecture; http://www.ensembl.org/Homo_sapiens/Variation/Population?db=core;r=7:22726127-22727127;v=rs1800796;vdb=variation;vf=729516870, accessed on 13 March 2024). Only *IL6* rs1800796, revealed by Tosa et al. as a genetic factor predisposing to keloids in Japanese individuals, potentially showed variation across populations, emphasizing the complexity of genetic influences on keloid formation and the necessity of considering diverse genetic backgrounds in such studies. In their study across two separate groups of healthy subjects, the frequencies of the keloid risk allele (rs1800796: −572G) were 16.5% and 17.3%, separately, and in two separate groups of patients with keloids, the values were 27.4% and 26.5%, separately [13].

According to the 1KG Project, the frequency of rs1800796 −572G is 17.8% in the Japanese population, 28.2% for Han Chinese from Beijing, 21.4% for Han Chinese from Southern China, and 17.7% for individuals from the Dai minority. The reference to data from the 1KG Project regarding China is particularly significant in the context of a study published in 2017 by Zhu et al., who analyzed the association of *IL6* polymorphisms rs1800796 and rs1800795 with susceptibility to keloid scar formation among Han Chinese from Jiangsu Province, located in the central part of China’s eastern coast [14]. In this study, the frequencies of the −572G and −174C alleles of the *IL6* gene were 29.9% and 35.2%, respectively, in the control group and 35.9% and 33.3%, respectively, in the study group. Zhu et al.’s study found a statistically significant difference in the frequency of the −572G > C polymorphism between the groups, confirming earlier observations by Tosa et al. in the Japanese population. The association of the rs1800796 polymorphism with the predisposition to keloid scar formation was also confirmed in 2019 by researchers in Egypt, who reported that the frequency of the risk allele (−572G) in patients with keloid scars in their country was as high as 67.5%, whereas it was less than half at 26.7% in the control group [17]. However, the results of this last report should be interpreted with caution as the study group consisted of only 60 patients and the control group of just 30 individuals, which highlights the importance of sample size and demographic variation in genetic studies related to keloid susceptibility.

In contrast to the Japanese groups studied by Tosa et al. [13], in our Polish study groups, including patients with keloids and healthy newborns serving as a control group, differences were found for each of the three *IL6* promoter polymorphisms. Of note, previously, healthy newborns have been widely used as control groups in similar association studies [36,37,38], providing representative samples of the general population [39]. Additionally, association studies using newborns as controls might also have advantages due to the exclusion of confounding environmental influences such as disease or lifestyle [40]. Moreover, both the study and control groups consisted of individuals born in Western Pomerania in Poland and are descendants of people who arrived in this region after World War II from nearly all regions of the former Second Polish Republic [41,42]. Therefore, contemporary residents of Polish Western Pomerania are considered a representative sample of the Polish population in genetic epidemiological studies [43,44]. Random selection of newborns born in one of the Szczecin hospitals for the control group further minimized the risk of population error and stratification [44].

It is important to note the significant differences related to ethnic origin between the frequency of rs1800796 −572G > C in Poles compared to the data available from the above-cited publications on Japanese, Chinese, and Egyptians [13,14,17]. In our study, the frequency of the −572G allele among patients with keloid scars was 89.5%, and it was as high as 94.0% in the control group of newborns. These values are very close to the frequency of this variant in Poles (94.4%) reported by Maculewicz et al. [45] and its frequency in other European countries, such as Finland (94.9%), the United Kingdom (96.2%), Spain (94.9%), Italy (94.9%), and the CEU population (95.5%; residents of Utah with ancestors from Northern and Western Europe), as analyzed in the 1000 Genomes project. (http://www.ensembl.org/Homo_sapiens/Variation/Population?db=core;r=7:2272612722727127;v=rs1800796;vdb=variation;vf=729516870, accessed on 13 March 2024). These findings highlight the significant genetic variability and the impact of ethnic background on the prevalence of specific alleles associated with keloid formation. The high frequency of the −572G allele in the Polish population and other European populations suggests a limited role of this polymorphism in keloid susceptibility within these ethnic groups, contrasting with its reported significance in Japanese, Chinese, and Egyptian populations.

Unlike Tosa et al. [13], who only identified reference genotypes for the *IL6* polymorphisms rs1800797 and rs1800795 among Japanese, we conducted an analysis of linkage disequilibrium for each possible pair of polymorphisms, namely rs1800797-rs1800796, rs1800797-rs1800795, and rs1800796-rs1800795. We then reconstructed a haplotype composed of three alleles: rs1800797-rs1800796-rs1800795. For the three analyzed pairs of *IL6* promoter polymorphisms, we observed a very tight (almost complete) linkage disequilibrium for the polymorphisms at position −597 (rs1800797) and at position −174 (rs1800795). Since there are no data on the linkage disequilibrium of these three *IL6* polymorphisms in Poles in the literature, we conducted such an analysis for a European-derived group from the 1000 Genomes project using a free bioinformatics tool available online (https://ldlink.nci.nih.gov/r, accessed on 13 March 2024). Our analysis, based on an evaluation of a group of 503 individuals from Finland, the United Kingdom, Spain, Italy, and the CEU population, confirmed this significant and very tight linkage disequilibrium between rs1800797 and rs1800795 (D` = 1.0, R^2^ = 0.9717). Three biallelic polymorphisms can create up to 8 haplotypes, and we did not find the A-C-G haplotype among 372 possible haplotypes (186 individuals × 2 haplotypes per person). The frequencies of the haplotypes A-C-C (H2), G-C-C (H4), and G-G-C (H7) were less than 2%; thus, in further analysis, we treated them collectively as rare haplotypes. In these analyses, the reference haplotype was taken to be G-G-G (H1), which had the highest frequency (52.9%). Since no publications regarding the distribution of rs1800797-rs1800796-rs1800795 haplotypes in the Polish population were found in the literature, we decided to perform a similar analysis for the European-derived population from the 1KG Project (503 individuals × 2 haplotypes = 1006 haplotypes) using the same online tool. Among the 8 possible haplotypes in these individuals, only 4 haplotypes were present, namely A-G-C (40.7%), G-C-G (4.8%), G-G-C (0.8%), and G-G-G (53.7%), and their frequencies were very similar to the frequencies of those haplotypes in our studied group of Poles. This comparison highlights the significance of genetic background in the study of polymorphisms and their associated risks or conditions. It also underscores the importance of haplotype analysis in understanding the genetic predisposition to diseases, such as keloids, within different populations.

In the classical signaling pathway, interleukin-6 first binds to the alpha subunit of its receptor (UNIPROT: IL-6RA, P08887; alternative name: gp80; antigen CD126), an 80-kDa transmembrane protein. Subsequently, both proteins together bind to the beta subunit of the receptor (IL-6RB; gp130; CD130), a transmembrane protein with a molecular mass of 130 kDa, forming a stable complex (effectively a hexamer composed of two ligand molecules, two IL-6RA subunits and two IL-6RB subunits). Only then is further IL-6 signal transmission possible, which occurs via the activation of three intracellular pathways: the JAK/STAT (Janus kinase/signal transducer and activator of transcription) pathway regulating the expression of several genes leading to the induction of differentiation, cell growth, and survival; the PI3K/AKT (phosphoinositide 3-kinase/protein kinase B) pathway associated with cell survival; and the MAPK (mitogen-activated protein kinase) pathway that triggers the transcription of genes related to growth, division, and cell differentiation, as well as the production of acute-phase proteins and immunoglobulins. The membrane-bound receptor for IL-6, composed of both subunits, is only present in a few cell types, including macrophages, neutrophils, CD4^+^ T cells, podocytes, and hepatocytes, so only these cells can directly respond to interleukin-6 [33]. However, trans-signaling is an alternative to the classic IL-6 signaling pathway and allows for the modulation of functions of a wide range of target cells (including cancer cells, neurons, or osteoclasts that do not have the membrane-bound receptor composed of alpha and beta subunits). In this mechanism, sIL-6RA, which comprises the extracellular domain of IL-6RA and retains the ability to bind IL-6 comparable to the membrane-bound form of the receptor, plays a key role. Thus, the IL-6/sIL-6RA complex can bind and activate the IL-6RB protein present in the membranes of many types of cells. In humans, sIL-6RA is produced as a result of proteolysis catalyzed by metalloproteases, which is considered the primary mechanism for creating the soluble form of the receptor, or as a result of alternative splicing and translation of mRNA for IL-6RA lacking the transmembrane and cytosolic domains [33]. An additional factor influencing both classical signaling and trans-signaling, as well as modulating the end result of the activation of both signaling pathways, is the polymorphism *IL-6R* rs2228145 (1073A > C, p.Asp358Ala) of the gene encoding the alpha subunit of the IL-6 receptor. Several studies have confirmed that the presence of the *IL-6R* c.1073C allele is associated with an increased concentration of sIL-6RA in the serum [46,47,48,49,50].

The results of the only study published to date regarding the analysis of the relationship between the polymorphism rs2228145 and keloid scars among Japanese subjects suggest that this polymorphism is not a predisposing factor for this disease [13]. In our study, we did not find that the polymorphism c.1073A > C was associated with a risk of keloids. Our confirmation of the results of the Japanese authors is particularly significant because, unlike the three promoter polymorphisms of the *IL6* gene, the frequencies of polymorphism rs2228145 among individuals of European descent and those from East Asia, including Japanese, are very similar. In the report by Tosa et al., the frequency of the *IL6R* c.1073C allele was 39.1% in Japanese patients with keloids and 42.3% in the control group of Japanese, while it was 39.0% among Poles with keloids and 36.5% in Polish newborns comprising the control group. Data from the 1000 Genomes Project indicate that the frequency of this *IL6R* variant among East Asians averages 32.3%, being lowest in the Dai minority in China (24.2%) and highest (37.9%) in Han Chinese from Beijing. Among individuals of European descent, the lowest frequency of the c.1073C allele was observed in Finns (25.8%), and the highest (41.2%) was noted in residents of Great Britain. The frequency of this allele in Poles is very close to the average (36.0%) of five European-derived populations from the 1000 Genomes project, ranging from 33.2% to 40.1%. This highlights the genetic variability across populations and the need for a nuanced understanding of genetic factors in disease predisposition [51,52,53,54,55,56].

Three major limitations of our study are (i) differences between various ethnic groups in the frequency of the analyzed *IL6* and *IL6R* variants, (ii) differences between ethnic groups in the occurrence of the clinical phenotype of keloids, and (iii) the relatively small size of our sample.

Analyses were conducted in a group of 186 Poles, including 86 individuals with keloids, while Tosa et al. [13] identified genotypes of the rs1800797, rs1800796, and rs1800795 polymorphisms of the *IL6* gene and rs2228145 of the *IL6R* gene in 615 Japanese, including 376 patients with keloids. Zhu et al. also analyzed the rs1800796 and rs1800795 polymorphisms in 470 Han Chinese, including 224 individuals diagnosed with keloids [14]. Only in the study of *IL6* rs1800796 among Egyptians was the group size (90 adults, including 60 patients with keloid scars) much smaller than in our report [17]. Using Open Epi (www.openepi.com, accessed on 13 March 2024), a free open-source software for statistical analysis, we calculated the minimum sample size for the polymorphism rs1800796 (using the dominant inheritance model for the −572C allele) to achieve 80% statistical power and a 5% type I error rate (α), assuming a ratio of the control group to the keloid group of 1.16 (100/86) and that the percentages of individuals with at least one −572C allele was the same as that in our study (i.e., 12.0% in the control group and 18.6% in the keloid group). Under these assumptions, the calculated confidence interval for the required (minimum) sample size ranged from 918 to 983 individuals, including between 425 to 455 patients with keloid scars.

## 4. Materials and Methods

### 4.1. Keloid Patients and Control Subjects

Both the studied group of patients with keloids and the control group have been described in detail previously [20,21]. All subjects in the study and control groups were Poles of European descent. The study was conducted in accordance with the latest Declaration of Helsinki (2013) and was approved by the bioethics committee at the Pomeranian Medical University in Szczecin, Poland. Patients provided informed consent, and parental informed consent was obtained for newborn controls.

### 4.2. Genetic Analyses

Genomic DNA was extracted either from peripheral blood leukocytes (keloid patients) or from umbilical cord blood leukocytes (newborn infants) using a commercially available DNA isolation kit (QIAamp Blood DNA Mini Kit, QIAGEN, Hilden, Germany). For the analysis of the *IL6* rs1800797, rs1800796, and rs1800795 polymorphisms, a polymerase chain reaction-restriction fragment length polymorphism (PCR/RFLP) method was applied using the following primer pair: forward, 5′-CAACCTCCTCTAAGTGGGCTGAA-3′; and reverse, 5′-CAATCAGCCCCACCCGC-3′ (TIB MOL BIOL, Poznań, Poland). The *IL6* amplicons (600 base pairs in length) were subsequently digested with FokI, SsiI (AciI), or Hin1II (NlaIII) restriction enzymes (FastDigest versions; Thermo Scientific, Waltham, MA, USA) for the identification of rs1800797, rs1800796, or rs1800795 genotypes, respectively. For the *IL6* rs1800797 (−597G > A) polymorphism, the PCR product was cut using FokI into fragments of 471 base pairs (bp) and 129 bp in the presence of the −597A allele or remained uncut in the presence of the −597G allele. For the *IL6* rs1800796 (−572G > C) polymorphism, the PCR product was cut using SsiI (AciI) into fragments of 456, 90, 51, and 3 bp in the presence of the −572G allele or into fragments of 546, 51, and 3 bp in the presence of the −572A variant. For the *IL6* rs1800794 (−174G > C) polymorphism, the PCR product was cut using Hin1II into fragments of 394, 177, 29, and 3 bp in the presence of the −174G allele or into fragments of 394, 122, 55, and 29 bp in the presence of the −174A variant. Restriction products in each case were electrophoretically separated and visualized with staining (using Midori Green; Nippon Genetics Europe, Duren, Germany) in 3% agarose gels. To verify the results, DNA samples of 10 randomly chosen newborns were sequenced. The *IL6R* rs2228145 polymorphism was also identified using sequencing, and amplification of an *IL6R* sequence that was 429 bp in length, including the rs2228145 polymorphism, was performed with PCR using 5′-AAATGGCCTGTTGGTTGG-3′ as the forward primer and 5′-CACCTACTATTATGCCAAGCCGT-3′ as the reverse primer. Both *IL6R* and *IL6* PCR products were purified using exonuclease I and alkaline phosphatase (FastAP Thermosensitive; ThermoFisher Scientific, Waltham, MA, USA) according to the manufacturer’s procedures. The products were sequenced (using BigDye^®^ Terminator v3.1 Cycle Sequencing Kits; Applied Biosystems, Life Technologies Polska, Warsaw, Poland). Electrophoresis and analyses were performed according to the manufacturer’s procedures (using an ABI PRISM 3100-Avant machine; Data Collection Software v2.0, Sequencing Analysis Software v5.4; Applied Biosystems). In each case, the results of *IL6* genotyping obtained with sequencing were as expected based on the PCR-RFLP analysis. All DNA samples were genotyped in a blind manner, i.e., the samples were anonymously labeled by one person and then genotyped by a second person.

### 4.3. Statistical Analyses

Possible divergence of *IL6* rs1800797, rs1800796, and rs1800795 or IL6R rs2228145 genotype frequencies from Hardy–Weinberg equilibrium was assessed using a *χ*^2^ test in keloid patients, newborn controls, and the combined group. The differences in *IL6* or *IL6R* allele frequencies between groups were also assessed using *χ*^2^ tests. The *IL6* and *IL6R* genotype frequencies were compared between keloid patients and newborn infants using logistic regression with adjustment for sex in additive, dominant, or recessive modes of inheritance for the risk (minor) allele. Linkage disequilibrium between pairs of *IL6* polymorphisms (i.e., rs1800797-rs1800796, rs1800797-rs1800795, and rs1800796-rs1800795) were analyzed using the “genetics” package of the R statistical platform. The reconstruction of a haplotype composed of these three *IL6* polymorphisms (i.e., rs1800797-rs1800796-rs1800795) was performed using the haplo.em function of the R package “haplo.stats”. The differences in *IL6* haplotype distribution between both groups were assessed in R using sex-adjusted logistic regression in additive, dominant, or recessive modes of inheritance for the haplotype of interest in comparison to the reference haplotype. The R statistical platform provides a free software environment for statistical computing and graphical analyses (ver. 2.11.1, R Foundation for Statistical Computing, Vienna, Austria, http://www.R-project.org). The remaining calculations were performed using a data analysis software system (Dell Statistica, version 13. Dell Inc. 2016, http://software.dell.com accessed on 13 March 2024, Round Rock, TX, USA). Two-tailed tests with *p* < 0.05 were considered statistically significant.

## 5. Conclusions

The rs1800797, rs1800796, and rs1800795 polymorphisms in the promoter of the *IL6* gene coding for interleukin-6 and the rs2228145 polymorphism in the *IL6R* gene coding for the alpha subunit of the IL-6 receptor did not predispose to the occurrence of keloids in the studied group of Poles. Furthermore, *IL6* promoter haplotypes were also not associated with an increased risk of keloids in Polish patients. This highlights the complex nature of keloid formation, suggesting that other genetic or environmental factors may play a more significant role in keloid susceptibility in this population.

## Figures and Tables

**Table 1:** Association analyses of *IL6* rs1800797, rs1800796, and rs1800795 and *IL6R* rs2228145 genetic polymorphisms with keloids in Polish patients.

Polymorphism (Chromosomal Location ^a^)	Allele ^b^	Distribution of Alleles, n (%)		*p*	Distribution of Genotypes, n (%)						*p*	*p*_A_	*p*_R_	*p*_D_
	(1/2)	Keloid Patients	Control Group		Keloid Patients (n = 86)			Control Group (n = 100)						
		1/2	1/2		1;1	1;2	2;2	1;1	1;2	2;2				
rs1800797 (7:22726602)	G/A	102/70 (59.3/40.7)	123/77 (61.5/38.5)	0.665	32 (37.2)	38 (44.2)	16 (18.6)	39 (39.0)	45 (45.0)	16 (16.0)	0.893	0.865	0.794	0.963
rs1800796 (7:22726627)	G/C	154/18 (89.5/10.5)	188/12 (94.0/6.0)	0.115	70 (81.4)	14 (16.3)	2 (2.3)	88 (88.0)	12 (12.0)	0 (0.0)	0.206	- ^c^	- ^c^	0.212
rs1800795 (7:22727026)	G/C	105/67 (61.0/39.0)	125/75 (62.5/37.5)	0.773	33 (38.4)	39 (45.3)	14 (16.3)	41 (41.0)	43 (43.0)	16 (16.0)	0.933	0.946	0.824	0.942
rs2228145 (1:154454494)	A/C	105/67 (61.0/39.0)	127/73 (63.5/36.5)	0.626	31 (36.0)	43 (50.0)	12 (14.0)	40 (40.0)	47 (47.0)	13 (13.0)	0.858	0.334	0.977	0.181

^a^ Single nucleotide polymorphism location was indexed to NCBI build 38 (GRCh38.p13). ^b^ Alleles 1 and 2 were defined as the major and minor (rarer) alleles, respectively. *p*—significance values for chi^2^ 2 × 2 table (alleles) or for chi^2^ 2 × 3 table (genotypes). *p*_A_, *p*_R_, or *p*_D_—significance values for sex-adjusted logistic regression in additive, recessive, or dominant modes of inheritance for the minor allele (allele 2), respectively. ^c^ The analysis was carried out only for the dominant mode (1;1 vs. 1;2+2;2) due to the very low frequency of 2;2 homozygotes.

**Table 2:** Association analyses of *IL6* haplotypes with keloids in Polish patients.

Haplotype	Whole Group *, n (%)	Keloid Patients, n (%)	Control Group n (%)	*p*_A_	*p*_R_	*p*_D_
H1 (G-G-G)	197 (53.0) H1	87 (50.6)	110 (55.0)	Reference haplotype		
H2 (A-G-C)	133 (35.8) H2	59 (34.0)	74 (37.0)	0.927	0.806	0.974
H3 (G-C-G)	24 (6.4) H3	12 (6.9)	12 (6.0)	0.235	-	0.287
H4 (A-G-G)	9 (2.4) H4	6 (3.6)	3 (1.5)	0.287	0.484	0.269
H5 (A-C-C) ^#^	5 (1.3)	5 (3.1)	0 (0.0)			
H6 (G-G-C) ^#^	3 (0.8)	2 (1.4)	1 (0.5)			
H7 (G-C-C) ^#^	1 (0.3)	1 (0.4)	0 (0.0)			
H8 (A-C-G)	0 (0.0)	0 (0.0)	0 (0.0)			
H2+H4+H7	9 (2.4)	8 (4.9)	1 (0.5)	0.205	-	0.222

* The whole group consisted of keloid patients and control newborns. ^#^ Rare haplotype with a frequency less than 2% in the whole group consisting of keloid patients and control newborns. *p*_A_, *p*_R_ i *p*_D—_sex-adjusted *p* values in the regression analysis for individual haplotypes in the additive, recessive, and dominant inheritance models in regard to the reference haplotype (H1 haplotype).

## Data Availability

Data are contained within the article.
